# Promoting health-related cardiorespiratory fitness in physical education: A systematic review

**DOI:** 10.1371/journal.pone.0237019

**Published:** 2020-08-03

**Authors:** Miguel Peralta, Duarte Henriques-Neto, Élvio Rúbio Gouveia, Luís B. Sardinha, Adilson Marques

**Affiliations:** 1 CIPER, Faculdade de Motricidade Humana, Universidade de Lisboa, Lisboa, Portugal; 2 ISAMB, Faculdade de Medicina, Universidade de Lisboa, Lisboa, Portugal; 3 Departamento de Educação Física e Desporto, Universidade da Madeira, Funchal, Portugal; 4 Madeira Interactive Technologies Institute, LARSyS, Funchal, Portugal; Instituto Politecnico de Viana do Castelo, PORTUGAL

## Abstract

**Background:**

This article aimed to systematically review the contribution of physical education (PE) classes to improve cardiorespiratory fitness (CRF) in children and adolescents; and to define potentially relevant factors for promoting CRF in PE classes.

**Methods:**

Studies were identified from searches in ERIC, PubMed, SPORTDiscus, and Web of Science databases. Primary source articles, relating PE classes and CRF, published up to July 2019 in peer-reviewed journals were eligible for inclusion. Specific inclusion criteria were: (a) having cross-sectional or longitudinal and observational or interventional study designs; (b) targeting school-aged children or adolescents; (c) measuring CRF, heart rate or CRF test results as an outcome; (d) having statistical analyses of the CRF, heart rate or CRF test results outcomes reported; (e) focusing on PE classes or PE interventions that did not extended time or frequency of the classes; and (f) published in English, French, Portuguese, or Spanish.

**Results:**

A total of 24 studies met the inclusion criteria. Overall, 10 studies have found a neutral effect of PE classes in students’ CRF, eight studies found that PE indeed contributed to the improvement of CRF and six studies revealed mixed findings, when PE classes where controlled for others variables (e.g. body mass index, intensity). Higher intensity PE classes consistently demonstrated contributions to improving students’ CRF.

**Conclusion:**

Review findings suggest that PE classes can contribute to the improvement of students’ CRF. Intensity, age and weight status were identified as potentially relevant factors for promoting CRF in PE classes. To improve CRF, higher intensity classes should be provided.

## Introduction

Cardiorespiratory fitness (CRF) mirrors the overall capacity of the cardiovascular and respiratory systems [1]. It is considered as an important health variable, which is associated with several risk factors for cardiovascular diseases independent of socio-demographic factors, diet, and physical activity [2, 3]. Furthermore, CRF is suggested to be a significant risk factor to include in the assessment of the metabolic syndrome for children and adolescents [4]. Hereby, the study of this variable and its associations to health is widely recognized as essential both among adults and youth.

The school setting gives youth the opportunity to be physically active, mainly through physical education (PE) classes [5]. For this reason, the school system is viewed as an important means of promoting physical activity and health among children and adolescents. When performed appropriately and incorporated as one component of a broad and holistic health education programme, fitness monitoring in PE serve as a valuable part of the curriculum and play a role in supporting healthy lifestyles and physical activity [6].

It has been suggested that PE classes may play a significant role in CRF development [7–9] and monitoring [10], as there are a number of field tests available that allow whole school classes to be assessed in one session [11, 12]. Therefore, PE teachers have several quality tools to assess the students’ CRF. Notwithstanding, evidence regarding the contribution of PE classes for the development of CRF in children and adolescents is inconsistent [13, 14] and most studies examine school-based physical activity intervention programs [15] instead of curricular PE.

Although the school setting and PE classes offer a platform that might help for improving [7, 8] and monitoring [10] of CRF, recent studies suggest that in the last decades CRF appears to have declined in children and adolescents worldwide [16, 17]. Due to its importance, this evidence is of great concern. In order to begin reversing the decline in CRF, understanding how PE classes contribute to the improvement and maintenance of CRF in children and adolescents is vital. To the best of our knowledge, there is no study available that summarizes findings regarding the effect of PE classes on the CRF of students. Thus, the aims of this review were: (1) to summarize literature findings on the contribution of PE classes for improving CRF in children and adolescents; and (2) to define, based on this review, potentially relevant factors for promoting CRF in PE classes.

## Methods

### Study identification

Four relevant electronic databases (PubMed, ERIC, SPORTDiscus, and Web of Science) were comprehensively searched to identify peer-reviewed articles published up to July 2019. Definition of search terms was discussed among the authors. The identified search terms were: “physical education” AND cardiorespiratory OR cardiopulmonary OR cardiovascular OR endurance OR aerobic OR fitness OR PACER OR FitnessGram OR VO_2_ OR “physical condition” OR “physical aptitude”. Search terms were used in each database to identify potential articles with abstracts for review.

### Study selection and selection criteria

Primary source articles, relating PE classes and CRF, published up to July 2019 in peer-reviewed journals were eligible for inclusion. Specific inclusion criteria were: (a) having cross-sectional or longitudinal and observational or interventional study designs; (b) targeting school-aged children or adolescents; (c) measuring CRF, heart rate or CRF test results as an outcome; (d) having statistical analyses of the CRF, heart rate or CRF test results outcomes reported; (e) focusing on PE classes or PE interventions that did not extended time or frequency of the classes; and (f) published in English, French, Portuguese, or Spanish. Articles that did not meet all the inclusion criteria were excluded. Titles and abstracts of the retrieved articles were independently assessed for eligibility for inclusion by two authors (AM, MP). Duplicates from the electronic database search were deleted. Full texts of all eligible articles were retrieved, and other possible relevant studies were searched in the references of those articles. Two authors (AM, MP) reviewed the text of potential studies, and decisions to include or exclude studies in the review were made by consensus.

### Data extraction and harmonization

Based on the PRISMA statement [18] a data extraction form was applied. Relevant data was extracted from manuscripts by one author (MP); coding was verified by two other authors (AM, ERG). Divergences were discussed among authors and solved. Data extracted included study design, sample size, age, country, content of PE / intervention, outcome measure, method and main findings. Outcome measures were either a direct (e.g. VO_2_max) or indirect measure (e.g. number of laps) of CRF, or heart rate during exercise. Main findings are presented as a description of the contribution of PE classes to the CRF.

### Study quality and risk of bias

The Quality Assessment Tool for Observational Cohort and Cross-Sectional Studies [19] was used to appraise risk of bias (study quality). This tool comprises a 14 item checklist for longitudinal studies, while for cross-sectional studies only 11 items could be applied. According to the criteria, each longitudinal and cross-sectional study was rated either good (when meeting 10–14 and 8–11 criteria, respectively), fair (when meeting 5–9 and 4–7 criteria, respectively), or poor (when meeting 1–4 and 1–3 criteria, respectively). Study quality was assessed by two researchers (AM, MP) independently and discrepancies were discussed and solved by agreement.

## Results

### Literature search

**Fig 1:**
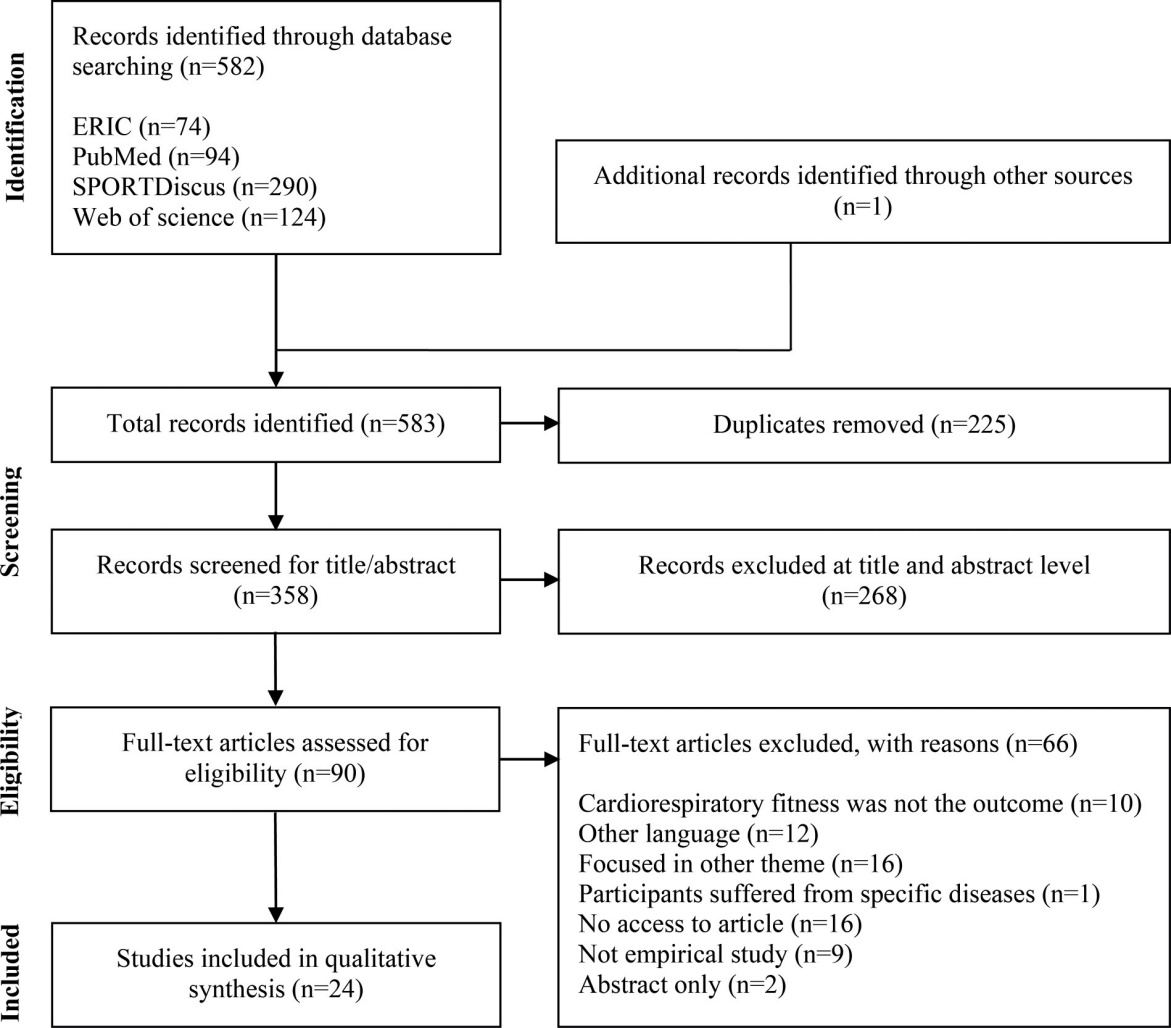
Flow diagram of studies.

Fig 1 presents the flow diagram of studies through the systematic review process. The systematic literature search identified a total of 582 studies. Additionally, one study was identified through a manual search and added to the review process. Out of these 583 studies, 225 were duplicated and thus removed, resulting in a total of 358 studies for title and abstract screening. After excluding studies at the title and abstract screening (n = 268), 90 studies were eligible for full-text reading and 66 were excluded with reasons. Therefore, a total of 24 studies were identified as relevant and included in this review.

### Risk of bias of included studies

Risk of bias of included studies was assessed by the Quality Assessment Tool for Observational Cohort and Cross-Sectional Studies [19] and is presented in Table 1. Most studies (19 out of 24) were classified as ‘fair’, one study received a ‘poor’ classification, and the other four studies were considered ‘good’.

**Table 1:** Risk of bias of included studies.

Author	1	2	3	4	5	6	7	8	9	10	11	12	13	14	Total
Cumming et al., 1969	N	Y	CD	Y	N	Y	Y	Y	Y	N	Y	N	CD	N	Fair
Crowhurst et al., 1993	Y	Y	CD	Y	N	Y	NA	Y	Y	NA	Y	N	NA	N	Fair
Strand & Reeder, 1993	Y	Y	CD	Y	N	Y	NA	CD	N	NA	Y	Y	NA	N	Fair
Baquet et al., 2001	Y	Y	CD	Y	N	Y	Y	Y	Y	Y	Y	N	CD	N	Fair
Baquet et al., 2002	Y	Y	CD	Y	N	Y	Y	Y	Y	Y	Y	N	CD	N	Fair
Koutedakis & Bouziotas, 2003	Y	Y	N	Y	Y	Y	NA	Y	Y	NA	Y	N	NA	N	Good
Beets & Pitetti, 2005	N	Y	CD	Y	N	Y	NA	N	Y	NA	Y	N	NA	N	Fair
Fairclough & Stratton, 2005	Y	Y	N	Y	N	Y	NA	Y	Y	NA	Y	N	NA	N	Fair
Fairclough & Stratton, 2006	Y	Y	CD	Y	N	Y	NA	Y	Y	NA	Y	N	NA	N	Fair
Laurson et al., 2008	Y	Y	CD	Y	N	Y	NA	Y	Y	NA	Y	N	NA	N	Fair
Pelclova et al., 2008	Y	Y	CD	N	N	Y	NA	N	Y	NA	Y	N	NA	N	Fair
Gallotta et al., 2009	Y	Y	N	Y	N	Y	Y	N	Y	N	Y	N	CD	N	Fair
Camhi et al., 2011	Y	Y	CD	Y	N	Y	Y	Y	Y	Y	Y	N	CD	N	Fair
Ramirez Lechunga et al., 2012	Y	Y	CD	Y	N	Y	Y	Y	Y	N	Y	N	CD	N	Fair
Lucertini et al., 2013	Y	Y	N	Y	N	Y	Y	Y	Y	N	Y	N	CD	N	Fair
Reed et al., 2013	Y	Y	N	Y	N	Y	Y	Y	Y	Y	Y	N	CD	Y	Good
Bendikson et al., 2014	Y	Y	CD	Y	N	Y	Y	Y	Y	N	Y	N	CD	N	Fair
Rengasamy et al., 2014	Y	Y	CD	Y	N	N	Y	N	N	N	Y	N	CD	N	Fair
Erfle & Gamble, 2015	Y	Y	Y	Y	N	Y	Y	Y	Y	N	Y	N	N	Y	Good
Mayorga-Veiga & Viciana, 2015	Y	Y	CD	Y	N	Y	Y	Y	Y	N	Y	N	CD	N	Fair
Jarani et al., 2016	Y	Y	N	Y	N	Y	Y	Y	Y	N	Y	N	Y	Y	Good
Mayora-Veiga et al., 2016	Y	Y	CD	Y	N	Y	Y	N	Y	N	Y	N	CD	N	Fair
Andres, 2017	Y	N	CD	CD	N	Y	Y	N	CD	N	N	N	CD	N	Poor
Park et al., 2017	Y	Y	N	Y	N	Y	Y	Y	Y	N	Y	N	CD	N	Fair

Y, yes; N, no; CD, cannot determine; NA, not available.

### Study characteristics

Study characteristics are summarized in Table 2. Included studies presented several designs (intervention, observational cross-sectional and observational longitudinal), outcome measures (VO_2_max, heart rate, test result), and methods to asses CRF. Studies from 14 countries were included, most of them with a mixed sex sample.

**Table 2:** Characteristics of the included studies.

Characteristics	Number of studies
**Study design**	
Cross-sectional	7
Longitudinal	2
Intervention	15
**Outcome measured**	
VO_2_max / predicted VO_2_max	7
Heart rate	10
Distance covered	3
Test duration	6
Number of laps	1
**Methods used**	
Cycle ergometer protocol	2
Monitoring heart rate	10
PACER test	2
Multistage 20-meter shuttle run test	4
Yo-Yo Intermittent Recovery Level 1 Children’s test	1
Intermittent shuttle run test	1
1-mile run/walk test	2
1-km run/walk test	2
12-Minute Cooper’s Test	1
7-minute running test	1
Gas analyser	1
**Sample characteristics**	
** Country**	
Albania	1
Canada	1
Czech Republic	1
Denmark	1
England	2
France	2
Greece	1
Italy	2
Malaysia	1
Poland	1
Spain	3
South Korea	1
Ukraine	1
United States of America	7
** Sex**	
Boys and girls	19
Boys	2
Girls	3
** Age**	
Younger ages (6–12 years old)	6
Older ages (11–19 years old)	18
**Study quality**	
Poor	1
Fair	19
Good	4

PACER, progressive aerobic cardiovascular endurance run.

### Main findings

Table 3 shows the main findings and characteristics of the studies included in this review. Included studies ranged from 1969 to 2017, demonstrating that scientific interest in the contribution of PE to promote CRF is close to 50 years old. Overall, 10 studies have found a neutral effect of PE classes in students’ CRF, eight studies found that PE indeed contributed to the improvement of CRF and six studies revealed mixed findings, when PE classes where controlled for others variables (e.g. body mass index, intensity). Although 24 studies were included in this review some presented more than one relevant finding, therefore, 33 findings regarding the contribution of PE to the promotion of students’ CRF are presented. This resulted in 16 findings indicating that PE did contribute to the improvement of students’ CRF, whereas 14 findings point to a neutral effect and 3 findings suggesting that students’ CRF decreased during a given time period in a PE program. However, there was some heterogeneity in the study populations, as well as PE class characteristics included in this review that must be considered.

**Table 3:** Characteristics and main findings of the studies included in the systematic review.

Source	Study design, sample size, age	Country	Study quality	Outcome measure(s)	Method(s)	Content of PE / intervention	Main finding(s)
Cumming et al., 1969	Longitudinal, n = 89 (boys only), 6^th^ and 10^th^ graders	Canada	Fair	VO_2_max	Submaximal cycle ergometer protocol	Not specified	(±) No changes in the VO_2_max from September to June of the following year (nine months)
Crowhurst et al., 1993	Cross-sectional, n = 9 (girls only), M_age_ = 14.6 years	USA	Fair	(1) VO_2_max	(1) Incremental, maximal cycle ergometer protocol	Basketball and floor hockey	(±) The intensity of PE classes (in minutes exercised at >50% of VO_2_max) may not generally be sufficient for achieving an aerobic benefit
				(2) Heart rate			
					(2) Heart rate monitor during PE lessons		
Strand & Reeder, 1993	Cross-sectional, n = 55, age range = 12 to 13 years	USA	Fair	Heart rate	Heart rate monitor during PE classes	Team games (e.g. football, dodgeball), swimming and wrestling	(±) Students spend <50% of time in their assigned training zone (>60% of heart rate reserve)
Baquet et al., 2001	Intervention, n = 551 (52% boys), age range = 11 to 16 years	France	Fair	Distance covered	7-minute running test	Running	(+) High intensity PE (aerobic training) classes improves CRF
Baquet et al., 2002	Intervention, n = 345 (59% boys), age range = 11 to 16 years	France	Fair	Heart rate	Heart rate monitor during PE classes	Running, jumping	(+) High intensity PE classes (in % time spent >50%, 60% and 75% of heart rate reserve) may improve CRF
Koutedakis & Bouziotas, 2003	Intervention, n = 84 (boys only), M_age_ = 13.6 years	Greece	Good	VO_2_max	Multistage 20-meter shuttle run test	Team games (e.g. football, handball), swimming, athletics, tennis	(±) Students participating only in PE classes have lower levels of VO_2_max than students participating in PE classes and other extracurricular organised physical activities
Beets & Pitetti, 2005	Cross-sectional, n = 187(64% boys), age range = 14 to 19 years	USA	Fair	VO_2_max	PACER test	Team activities	(±) Students participating in PE classes have lower levels of VO_2_max than students participating in school-sponsored sports programs
Fairclough & Stratton, 2005	Cross-sectional, n = 122 (50% boys), age range = 11 to 14 years	England	Fair	Heart rate	Heart rate monitor during PE classes	Team games (e.g. football, softball), individual games (e.g. badminton, tennis), movement activities (e.g. dance, gymnastics) and individual activities (e.g. athletics, fitness)	(±) Students spent <50% of time in MVPA. Students participated in most MVPA during team games and the least during movement activities
Fairclough & Stratton, 2006	Cross-sectional, n = 68 (49% boys), age range = 11 to 14 years	England	Fair	Heart rate	Heart rate monitor during PE classes	Team games, individual games, gymnastic, dance	(±) Students spent <50% of time in MVPA
Laurson et al., 2008	Cross-sectional, n = 796 (53% boys), M_age_ = 16 years	USA	Fair	Heart rate	Heart rate monitor during PE classes	Team games (e.g. volleyball, ultimate frisbee), individual games (e.g. golf, dance), fitness activities (e.g. aquatics, bleachers)	(+) 71% of class time was spent in MVPA (>50% of maximum heart rate)
							(+) Fitness activities provided greater % of time above the lower heart rate threshold than individual and team games
Pelclová et al., 2008	Cross-sectional, n = 241 (girls only), M_age_ = 16.0 years	Czech Republic and Poland	Fair	Heart rate	Heart rate monitor during PE classes	Dance and aerobic dance	(+) Girls spent more than 50% of class time (aerobic dance classes) in MVPA (>60% of maximum heart rate)
Gallotta et al., 2009	Intervention, n = 152, age range = 11 to 12 years	Italy	Fair	Test duration	1-mile run/walk test	Pre-tumbling, rhythmic gymnastics, ball mini-games, dexterity circuits	(±) There were no significant differences in the 1-mile run/walk test results five months apart, for both control (regular PE classes) and intervention groups
Camhi et al., 2011	Longitudinal, n = 131 (girls only), M_age_ = 13.8 years	USA	Fair	Heart rate	Heart rate monitor during submaximal step test	Aerobic dance, football, walking/jogging, fitness activities (e.g. resistance training, circuit training), swimming, basketball, volleyball, recreational games	(+) Normal-weight and overweight girls enrolled in an eight months PE program showed improvement in fitness (decrease in stage 1 heart rate), as well as maintenance of these effects over the two next years
							(±) Obese girls showed no fitness improvements in response to the same PE program.
Ramirez Lechuga et al., 2012	Intervention, n = 84 (61% boys), age range = 15 to 18 years	Spain	Fair	VO_2_max	Portable gas analyser during multistage 20-meter shuttle run test	Running	(+) A eight weeks high intensity aerobic training program developed in PE classes improved students’ VO_2_max
							(±) During the same 8-week period, regular PE classes did not improved students’ VO_2_max
Lucertini et al., 2013	Intervention, n = 101, (50% boys), 3^rd^ to 5^th^ graders	Italy	Fair	VO_2_max	Multistage 20-meter shuttle run test	Basic motor skills, rhythm, coordination, endurance, strength, flexibility	(+) Specialist led and generalist teacher led PE classes increased primary school children’s VO_2_max during a six months period
Reed et al., 2013	Intervention, n = 470 (50% boys), 2^nd^ to 8^th^ graders	USA	Good	Number of laps	PACER test	Fundamental skills, multiactivity sport theme curriculum	(+) CRF of elementary school students participating in regular PE increased in an eight months period
							(–) CRF of middle school students participating in regular PE decreased in an eight months period
Bendiksen et al., 2014	Intervention, n = 91 (55% boys), age range = 8 to 9 years	Denmark	Fair	(1) Heart rate	(1) Heart rate monitor during YYIR1C	Team games (e.g. football, unihockey), individual games (e.g. walking, parkour), Nintendo Wii Boxing, Nintendo Wii Tennis	(±) Students participating in regular PE classes did not improve CRF (distance covered and % of maximal heart rate) in a six weeks period
				(2) Distance covered	(2) YYIR1C		
							(+) Students participating in high intensity PE classes improved CRF (distance covered and % of maximal heart rate) in a 6 weeks period
Rengasamy et al., 2014	Intervention, n = 173 (50% boys), M_age_ = 16 years	Malaysia	Fair	Distance covered	12-Minute Cooper’s Test	Circuit training	(+) A 10-week fitness program implemented within PE classes enhanced the students’ CRF
Erfle & Gamble, 2015	Intervention, n = 10206 (50% boys), 6^th^ to 8^th^ graders	USA	Good	Test duration	1-mile run/walk test	Not specified	(±) Students participating in regular PE classes did not improve CRF during one school year
Mayorga-Veiga & Viciana, 2015	Intervention, n = 178 (58% boys), elementary and middle school children	Spain	Fair	Test duration	Multistage 20-meter shuttle run test	Fitness activities (e.g. circuit training, multi-jumps), team games	(–) CRF of middle school students participating in regular PE decreased in eight weeks period
							(+) CRF of elementary and middle school students with low CRF participating in high intensity PE classes (fitness program) improved in an eight weeks period
Jarani et al., 2016	Intervention, n = 767 (52% boys), 1^st^ and 4^th^ graders	Albania	Good	VO_2_max	Intermittent shuttle run test	Throwing/catching, rhythm activities (e.g. dance), fitness activities, tumbling / gymnastics	(+) Exercise (fitness) and game-oriented PE classes improved children’s CRF and have greater effect in improving CRF than other PE classes
Mayorga-Veiga et al., 2016	Intervention, n = 111 (63% boys), M_age_ = 12.5 years	Spain	Fair	(1) Test duration	(1) Multistage 20-meter shuttle run test	Fitness activities (circuit training, multi-jumps), team games	(+) Students participating in high intensity PE classes (fitness program) improved CRF in a nine weeks period and maintained the improvements after four weeks detraining period
				(2) Heart rate	(2) Heart rate monitor during PE classes		
							(+) High intensity PE classes had >50% of time in MVPA
							(–) Students participating in regular PE classes decreased CRF in a nine weeks period
							(±) Regular PE classes had <50% of time in MVPA
Andres, 2017	Intervention, n = 100	Ukraine	Poor	Test duration	1-km run/walk test	Not specified	(±) No improvements in CRF from October to May of the following year (seven months)
Park et al., 2017	Intervention, n = 48 (50% boys), M_age_≈12 years	South Korea	Fair	Test duration	1-km run/walk test	Fitness activities (e.g. burpees, shuttle run)	(+) CRF of children participating in PE improved, while CRF of children not participating in PE decreased after an eight weeks period

CRF, cardiorespiratory fitness; PE, physical education; PACER, progressive aerobic cardiovascular endurance run; YYIR1C, Yo-Yo Intermittent Recovery Level 1 Children’s test; MVPA, moderate-to-vigorous physical activity; M_age_, mean age.

(+) Found a positive effect of PE on students’ CRF (positive changes in CRF or ≥50% of time in MVPA) (n = 16).

(±) Found a neutral effect of PE on students’ CRF (no changes in CRF or <50% of time in MVPA) (n = 14).

(–) Students’ CRF decreased during a given time period in a PE program (n = 3).

Findings from younger students (n = 7) [13, 20–24], ranging from 6 to 12 years, showed mainly that participation in PE classes improved the students’ CRF [13, 21, 22], and in two of these studies the improvements were due to high intensity (whether fitness oriented or game oriented) PE classes [23, 24]. Notwithstanding, two other studies from this set concluded that PE had a neutral students’ CRF [20, 23]. On the other hand, findings from older students (n = 26), with a wider age range (approximately 11 to 19 years), were mixed. A total of 15 findings from 14 studies showed that PE had a neutral effect on students’ CRF (n = 15) [14, 22, 25–36]. While, 11 findings from nine studies reported that PE had a positive effect in children’s and adolescents’ CRF [26–28, 36–41].

Studies concluding that PE classes had a neutral effect on students’ CRF are supported by two main findings: (1) children and adolescents participating in PE classes did not improve or decreased their CRF during a given time period (n = 7) [14, 22, 26–29, 35]; and (2) PE classes did not provide sufficient intensity for achieving an aerobic benefit (n = 5) [28, 30, 31, 33, 34]. Besides these findings, two other studies found that students participating only in PE classes had lower CRF levels than their peers participating in school-sponsored sports programs [32] and in extracurricular organized physical activities [25].

Almost all studies (n = 10) with findings indicating that PE contributed to improving CRF in students are related with the intensity level of the classes. Six studies indicated that high intensity PE classes, involving fitness activities or aerobic training, improved the students’ CRF in a given time period [26–28, 37–39]. Furthermore, three other studies showed that high intensity and fitness oriented PE classes had more than 50% of time spent in moderate-to-vigorous physical activity (MVPA) [28, 40, 41]. Finally, one study identified fitness activities as the greatest provider of time in MVPA, compared with individuals games and team games [40].

One study [36], in which the analysis was divided into three groups according to body mass index classification, showed that: while normal-weight and overweight girls enrolled in PE showed improvements in fitness, as well as maintenance of these improvements; obese girls, enrolled in the same PE program, did not.

From the review of these results, three potential relevant factors for promoting CRF in PE classes were identified: students’ age, PE classes’ intensity, and students’ weight status.

## Discussion

This review summarizes literature findings from studies published up to July 2019 on the contribution of PE classes for promoting CRF in children and adolescents. Twenty-four studies were included and systematically reviewed. Overall, this review revealed that findings regarding the contribution of PE classes to the promotion of CRF are mixed. Several findings suggested that PE has a neutral effect on students’ CRF, while others reinforce its importance. However, higher intensity PE classes consistently demonstrated having a positive contribution in promoting students’ CRF. Additionally, some other potentially relevant factors for promoting CRF in PE classes were identified, such as age and weight status. Review findings are discussed accordingly to these factors.

All studies were focused on school-aged children, however, due to the wide age range of the studies’ populations, findings were organized in two age groups. This separation enabled some differences in findings between younger and older students to be found. While for older ages PE seems to be less effective in promoting students’ CRF [14, 22, 25–36], for younger ages almost all studies suggested that PE classes improved the students’ CRF [13, 21–24]. From a physiologic standpoint, CRF naturally increases as children grow-up. This increase is fairly linear in boys until later adolescence, whereas in girls it plateaus around age 13 [42, 43]. Furthermore, during the early stages of adolescence, participation in physical activity and the corresponding physical fitness begins to show some decline [44]. Thus, increasing CRF, related to body growth, occurring at younger ages and decreasing participation in physical activity in older students may explain why improvements in CRF for a given period of time are more frequently found in younger children and adolescents. Another possible reason for the apparently less effective contribution of PE to the improvement of CRF in older students is motivation. Motivation to participate in PE seems to decline in the late elementary and high school years [45, 46], possibly resulting in decreasing physical activity both during PE and in leisure time. Considering these findings, PE may have a bigger role to play in promoting older students’ CRF than it does in younger students, through providing MVPA opportunities.

Aerobic exercise has been shown to increase CRF by about 5–15% in youth [42, 47]. Additionally, improvements in CRF, involving structural and functional adaptations, as well as in the oxidative capacity of skeletal muscle occur with regular MVPA participation [42]. In this review, five studies reported that PE classes did not provide sufficient intensity for achieving an aerobic benefit [28, 30, 31, 33, 34] and thus, did not contribute in a consistent manner to promote students’ CRF. It is clear that CRF in youth increases with activity of sufficient intensity, leading to improvements in maximal stroke volume, blood volume, and oxidative enzymes after exercise [48]. Consequently, time spent in MVPA during PE classes should be monitored and adequate to promote health. Also, findings suggesting that PE has a positive contribution in improving CRF were mainly related to the intensity level of the classes. The majority of studies examined in this review involved intervention programs built to increase PE class intensity without increasing the number of classes or curricular time dedicated to PE [23, 26–28, 37–39, 41]. In fact, four of these studies reported that students participating in PE classes from the intervention programs increased their CRF levels, while students participating in regular PE classes, i.e. classes that were not part of the program, decreased or maintained their CRF levels [23, 26–28]. Considering the importance of CRF for health, the lack of intensity in PE classes is worrying. From a public health perspective, PE has the potential to provide the tools to face the current youth obesity and sedentary epidemic [49]. However, in order to effectively contribute to CRF and health, it is urgent to find strategies to increase the intensity of PE classes.

One study [36], examining whether an eight months PE program improved students’ CRF when considering body mass index categories, suggested that although normal-weight and overweight girls showed improvement in fitness and maintenance of these effects over the next two years, their obese peers did not. Physical activity and body mass index are inversely correlated in children and adolescents [50]. Also, studies of usual physical activity in children suggest that the overweight and obese are less active [51, 52] and have poorer fundamental movement skills than their normal-weight counterparts [53]. Mastering of fundamental motor skills is strongly related to physical activity in children and adolescents and is critical to fostering physical activity since these skills are the foundation for advanced and sport-specific movement [54]. Furthermore, a recent meta-analysis found that overweight and obese students were, respectively, 27% and 54% more likely to have school absenteeism than their normal weight peers [55]. All these factors may contribute to a greater ineffectiveness of PE programs among obese students. Therefore, PE should not only provide sufficient intensity to promote health, but also be based on developmentally appropriate motor activities to nurture self-efficacy and enjoyment and encourage ongoing participation in physical activity.

High quality PE together with appropriate approaches to fitness as part of health education programmes has been shown to promote fitness and healthy lifestyles [6]. On the other hand, when applied inappropriately and without context fitness monitoring can have the opposite result [56]. Therefore, mixed findings found in this systematic review may be due to the variations in the quality of PE. It is important that high quality PE is provided and promoted in schools, as it benefits not only students’ CRF, but also promotes future healthy lifestyles.

The present review has some limitations that should be acknowledged. Even though study bias was assessed according to their methodological quality [19], they were not weighted or ranked, thus, findings from studies with poorer quality and smaller sample sizes were given no less importance than other findings. Nonetheless, only one study was classified as ‘poor’ quality. Additionally, included studies had a wide publication date range, from 1969 to 2017, and were from different cultural and socioeconomic contexts, which could have implications on what PE classes represent as well as PE curricula. Also, 16 studies were not accessible to the authors. Grey literature research was not included. Despite the developed efforts for reducing publication bias by the authors, this should be taken into account.

Notwithstanding, to the best of our knowledge, this is the first systematic review focused on the contribution of PE classes or PE interventions that did not extend time or frequency of the classes to the improvement of students’ CRF. Furthermore, an extensive research strategy comprising four different databases and several keywords was used. Future research should continue to investigate the contribution of PE to the improvement of CRF and other fitness attributes, and examine which curricula offers the best opportunity to improve fitness, health and promote life-long physical activity behaviours.

## Conclusions

Review findings from the 24 included studies suggest that PE classes can contribute to the promotion of students’ CRF. Some potentially relevant factors for promoting CRF in PE classes were identified, such as intensity, age and weight status. Exercise intensity is essential to promote CRF and other health outcomes in youth [42, 57, 58], thus it is not surprising that higher intensity PE classes demonstrated improvements in CRF. Findings from studies of younger students more consistently reported improvements in CRF than findings from studies of older students. Therefore, as older students may be more vulnerable to decreasing physical activity levels [44], PE should be keen in providing tools and opportunities to improve and maintain CRF levels in these ages. Regarding weight status, overweight and obese students should be a priority concern, as they may have more difficulty in improving CRF than normal-weight peers. Given that CRF is an independent health predictor and that decrease of CRF is a global trend, more efforts should be done to promote CRF in PE classes. High quality PE is needed as it can be a successful strategy in improving CRF levels.

## Supporting information

S1 File (DOC)
